# Exploring placemaking in Boston’s low-threshold transitional housing locations

**DOI:** 10.1016/j.healthplace.2026.103634

**Published:** 2026-02-17

**Authors:** Joseph Silcox, Charlie Summers, Sofia Zaragoza, Patricia Case, Avik Chatterjee, Sarah Porter, Traci C. Green

**Affiliations:** a The Heller School for Social Policy & Management at Brandeis University, Waltham, MA, USA; b Department of Anthropology at Brandeis University, Waltham, MA, USA; c University of California, Berkeley, School of Public Health, Berkeley, CA, USA; d Northeastern University, Bouve College of Health Sciences, Boston, MA, USA; e Boston Medical Center, Boston, MA, USA; f Boston University, Boston University Chobanian & Avedisian School of Medicine, Boston, MA, USA; g Victory Programs, Inc, Boston, MA, USA; h Brown University Schools of Medicine and Public Health, Departments of Emergency Medicine and Epidemiology, Providence, RI, USA

## 1. Introduction

In 2014, the City of Boston abruptly closed substance use treatment and low-barrier housing services on Boston Harbor’s ‘Long Island’ due to structural concerns related to the island’s connecting bridge (Leverentz, 2022; Silcox, 2024). These closures displaced large numbers of unhoused people who use drugs (PWUD) into Boston’s South End (Leverentz, 2022; Mayer et al., 2024; Pittman, 2020), gradually forming a large ‘encampment’ at the intersection of Massachusetts Avenue and Melnea Cass Boulevard (hereafter, Mass and Cass). The encampment grew in size during the COVID-19 pandemic (Schwartz, 2022) and has since been the subject of ongoing political discussions due to the area’s open-air drug use and distribution, violence, and visible homelessness (Leverentz, 2022; Mayer et al., 2024; Pittman, 2020). In response to frequent media coverage and publicly communicated frustrations from South End residents, Boston’s current mayor, Michelle Wu, declared a ‘public health emergency’ at Mass and Cass in January of 2022. This declaration was eventually followed by an ‘Unlawful Camping Ordinance’ in October 2023, which resulted in the mandated relocation of encampment residents, as well as the removal of tents and other belongings from the area against encampment residents’ wishes (Wuthmann, 2023).

The situation at Mass and Cass is representative and follows patterns of similar attempts to clear homeless encampments in other major U.S. cities. Federal, state, and local governments increasingly impose sanctions on those living outdoors or in public spaces, forcing relocation or displacement (Ludden, 2024). Research highlights that these forced relocations, sometimes referred to as ‘sweeps’, are particularly dangerous for (PWUD), as they distance them from established support systems and increase risks of adverse events, such as fatal overdose (Collins et al., 2019). Some local and state governments hope to mitigate these risks through expanding existing social services or improving their shelter infrastructure. Following this lead, as part of efforts to remove unhoused people from Mass and Cass, the city of Boston implemented seven low-threshold, transitional housing (LTTH) sites across the city (Beaugard et al., 2024; Komaromy et al., 2023; Mayer et al., 2024; Chatterjee et al., 2025). Following an evidence-based, Housing First approach, abstinence from drug use was not required prior to placement at LTTH sites.

LTTH sites utilize principles commonly associated with Housing First models but further build on these principles through their implementation of specific harm reduction policies and practices. ‘Harm reduction’ is an approach to drug use rooted in grassroots activism from the 1970s-1980s, originally heavily tied to efforts to address the transmission of infectious diseases like hepatitis B and HIV (Inciardi and Harrison, 2000). A harm reduction approach focuses on reducing the negative effects of health behaviors without necessarily eliminating behaviors completely (Hawk et al., 2017). Today, harm reduction as it pertains to drug use aims to mitigate the consequences of drug use rather than demand total cessation of drug use itself (Mayer et al., 2024). Common harm reduction strategies include syringe exchange, overdose prevention and response, low-barrier medication assisted treatments and peer support. Boston’s LTTH sites aimed to provide high quality, low-barrier transitional housing alongside access to clinical, mental health and harm reduction services within LTTH sites LTTH sites attempted to ideologically incorporate harm reduction principles throughout their programming, in efforts to best meet clients where they were at with respect to their drug use and recovery.

Importantly, while these sites served the acute needs of people experiencing homelessness, they also became a political tool for justifying the rapid relocation of individuals from the Mass and Cass area. Not everyone displaced from the Mass and Cass area was offered a transitional housing placement following the massive encampment clearing (Mayer et al., 2024), but the possibility and promise of a placement was integral to the approval of the Unlawful Camping Ordinance (City of Boston, 2023). This conflation impacted some residents’ experiences in being placed at the sites, as some residents felt their placements were coerced, even as others appreciated or sought out LTTH placement (Bedford, 2024). Despite this, LTTH sites allowed many residents to experience stable, longer-term housing placements for the first time. These sites also provided a space where residents could more easily navigate barriers associated with obtaining permanent housing solutions (e.g., clearing warrants, acquiring identification cards, etc.) as well as take steps to address underlying health concerns (e.g., establishing healthcare or substance use support service). The combination of low-threshold and harm reduction services opened new opportunities for residents to create spaces of their own, allowing them to engage in ‘placemaking’ as they navigated transitions from conventional shelter environments or from the Mass and Cass encampment.

‘Placemaking’ refers to the process through which individuals “create quality places to live, work, play and learn” (Ellery et al., 2021; Pierce et al., 2011). Placemaking theory emphasizes the role of place in shaping personal identity (Wright, 1997), asserting needs and desires (Proshansky et al., 1983), reusing or repurposing space (Camerin, 2024), and resisting oppressive political narratives and systems (Toolis, 2017). The process of placemaking is an inherently networked process, shaped by socio-spatial relationships that connect individuals through shared physical space (Pierce et al., 2011). For example, ethnographic observations within unhoused communities by Douglas (2023) tie placemaking to politics of legitimacy and visibility, contending that placemaking among unhoused people plays a crucial role in asserting a right to permanent housing. Unhoused communities often engage in placemaking as a response to repeated displacement efforts, demonstrating that there is “someplace” for them to belong, rather than “no place”. However, research has also found that a lack of physical attachments or personally owned items (e.g., phones, clothing, or temporary shelter supplies like tents) among those who are unhoused may lead to a sense of belonging, as being together helps create meanings of ‘home’, separating these situations from physical place (Fathi et al., 2025). Conversely, unhoused individuals may have complicated relationships to physical places that become part of their struggle to be permanently housed. Further understanding placemaking efforts, particularly among unhoused communities, can provide meaningful insight into how placemaking can be used to shape social life within place (Camerin, 2024). Further, understanding the formal or informal applications of placemaking or how reuse of space might be practical (Lynch, 2022) can provide tangible outcomes for planners and policy-makers by providing legitimacy to how spaces may be used across a variety of different social contents. This is particularly applicable to LTTH sites, as these sites were often repurposed or reimagined spaces, transformed to support the acute and evolving needs of a previously unhoused population.

As low-threshold transitional housing options continue to develop in Massachusetts and across the country in response to chronic homelessness, there is a need to further explore how new residents within transitional housing sites come to interpret and develop a sense of ‘place’. How do transitional housing residents engage with ‘place’ to assert new claims about their identity? How do these placemaking efforts allow residents to express their desires for permanent housing? How does the practice of placemaking distinguish itself as unique within LTTH sites, in contrast to traditional shelters or street homelessness? In this paper, we observe how LTTH residents utilized placemaking strategies to cultivate non-stigmatized identities, express their desire for autonomy, and assert their right to housing, while also improving their overall health and wellness in the context of transitional housing spaces.

The current study focused on exploring and understanding the unique characteristics of LTTH locations in Boston through ethnographic observations and photographs collected from LTTH sites and residents. In this photo-ethnographic analysis, we focus on how LTTH residents make their living spaces meaningful, functional, and personal, highlighting stories and identities that have historically been rendered invisible. Through crafting their own privacy and security, personalizing their space, fostering self-protective and preventive health practices in their space, and constructing images of resistance, residents use place-making to legitimize their right to a home. Our analysis provides context into how residents engaged in place-making within these transitional housing spaces, allowing them to re-acquire skills needed to be successful for independent living and to reclaim identities.

## 2. Methods

### 2.1. Study design

The parent study was developed from formative rapid assessment work conducted at Mass and Cass which examined the initial encampment clearing processes and the development and implementation of the newly formed LTTH sites. The full study protocol details are reported elsewhere (please see Green et al., 2025). During the exploratory phase of our study, research staff conducted ethnographic observations through documenting field observations over the course of five to seven site visits. During these site visits, research staff took exploratory photographs of both the shared communal and outdoor spaces of each site they were permitted to enter. Photographs and ethnographic field notes were collected and stored securely in a shared study drive and were referenced throughout our analytic processes. Among the seven LTTH sites included in the study, six sites allowed study staff to photograph shared communal spaces. These data were then used to develop a comprehensive inventory (see Zaragoza et al., 2025 for additional details) that was designed to assess resident’s knowledge and engagement with harm reduction, clinical and other resources offered on site. For the current analysis, we focus on shared communal space photographs and ethnographic fields notes collected during the exploratory phase of our study and resident photographs and field notes collected following participation in our baseline survey.

### 2.2. Recruitment

The parent study used a multi-stage cohort design with extensive recruitment procedures that relied on purposive sampling to enroll residents of LTTH sites. Participants completed a comprehensive survey at baseline and again at 12-months. LTTH residents were eligible for enrollment in the study if they were: 1). 18 years or older 2). residing in one of the seven LTTH sites during baseline data collection or were previously enrolled in the formative rapid assessment study, and 3). reported using an illicit drug, in the past 30 days (not including cannabis). Recruitment of participants occurred in a variety of ways such as on-site, direct referrals from LTTH staff, and self-referral through exposure to study recruitment materials (e.g., flyers). Interested participants were screened for eligibility then consented by study research staff (for additional details on study recruitment procedures please see Green et al., 2025).

### 2.3. Survey & photographs

Research staff conducted a baseline survey with 107 individuals from September 2023 through February 2024. The survey asked residents living at the LTTH locations about resident experiences, drug use, and health changes related to relocation. Residents completed the surveys either on-site or at a nearby public location with research staff. Following successful completion of the baseline survey, all participants were asked if they would be willing to let research staff photograph their individual living spaces. Consent for this process was obtained through an additional written informed consent process where research participants could indicate what they felt comfortable being photographed. Research staff took all photographs on a secure study device and stored them in a shared drive to protect participants confidentiality and privacy. To further protect confidentiality, information provided on the survey was not linked to any participants’ photographs. If participants shared additional context about their photographs, research staff recorded these data in ethnographic field notes.

### 2.4. Sample & study setting

In total, 15 residents (14% of 107 survey participants) consented to having their private sleeping quarters photographed. The composition of resident rooms varied depending on the infrastructure of the LTTH site. Site A, a men’s shelter, consisted of connected cubicle sleeping spaces, open on one side and distributed throughout a wide room, with a large cafeteria-esque space near the entrance. Site B, a co-ed tiny house community, consisted of an enclosed outdoor space populated by “cottages” or tiny houses where residents lived alone or with a partner, as well as a community trailer for meals and interfacing with staff. Site C, a repurposed hotel, housed residents in individual rooms (sometimes with roommates) and offered a small hotel-style lobby for meals and socialization. Site D, a women-only site, was located on the fifth floor of a larger shelter, with each resident residing with at least one roommate. Site E was located on the second floor of a traditional emergency men’s shelter and had an open floor plan and no individual rooms. Site F, another repurposed hotel, closed prior to the second phase of the study, therefore only communal spaces were photographed. Resident living space photographs, in combination with photographs of their communal spaces, totaled 25 photographs taken from Site A, 26 from Site B, 11 from Site C, 19 from Site D, 3 from Site E, and 9 from Site F.

### 2.5. Data analysis

The research team inductively developed thematic codes by collectively reviewing communal and individual living space photographs to develop a codebook later refined through weekly coding meetings led by the first and fourth author. All study photographs were qualitatively coded in Dedoose Version 9.0.17 by three study team members (CT, CS, PC). Researchers coded specific features and items seen within communal and resident spaces such as cooking appliances, collections, decorations, harm reduction supplies, and creative tools. In the coding process, researchers looked for ways in which residents personalized and manufactured their space that reflected resident needs and identities. Themes and observations from coding were memo-ed and discussed during weekly team meetings (Charmaz, 2006). Whenever possible, coding was informed by field notes that included residents’ comments on their decision-making process when organizing their space. However, some photographs did not come with field notes as residents did not want to further expand on photographs. Others (such as photographs of common spaces) did not require dialogue with any specific residents. Throughout this analysis, researchers reflected on how their own identities such as race, gender, age and personal experiences of drug use or homelessness may influence the interpretation of the data (Campbell et al., 2021; May and Perry, 2014). This photo-ethnographic analysis of LTTH captured how residents and staff cultivated home-like, personalized environments within temporary living spaces. We use the theory of placemaking as a guiding theoretical framework to contextualize resident’s use of space among politics of legitimacy, agency, and belonging. All project activities were reviewed and approved in accordance with the Brandeis University Institutional Review Board.

## 3. Results

**Fig. 1.**
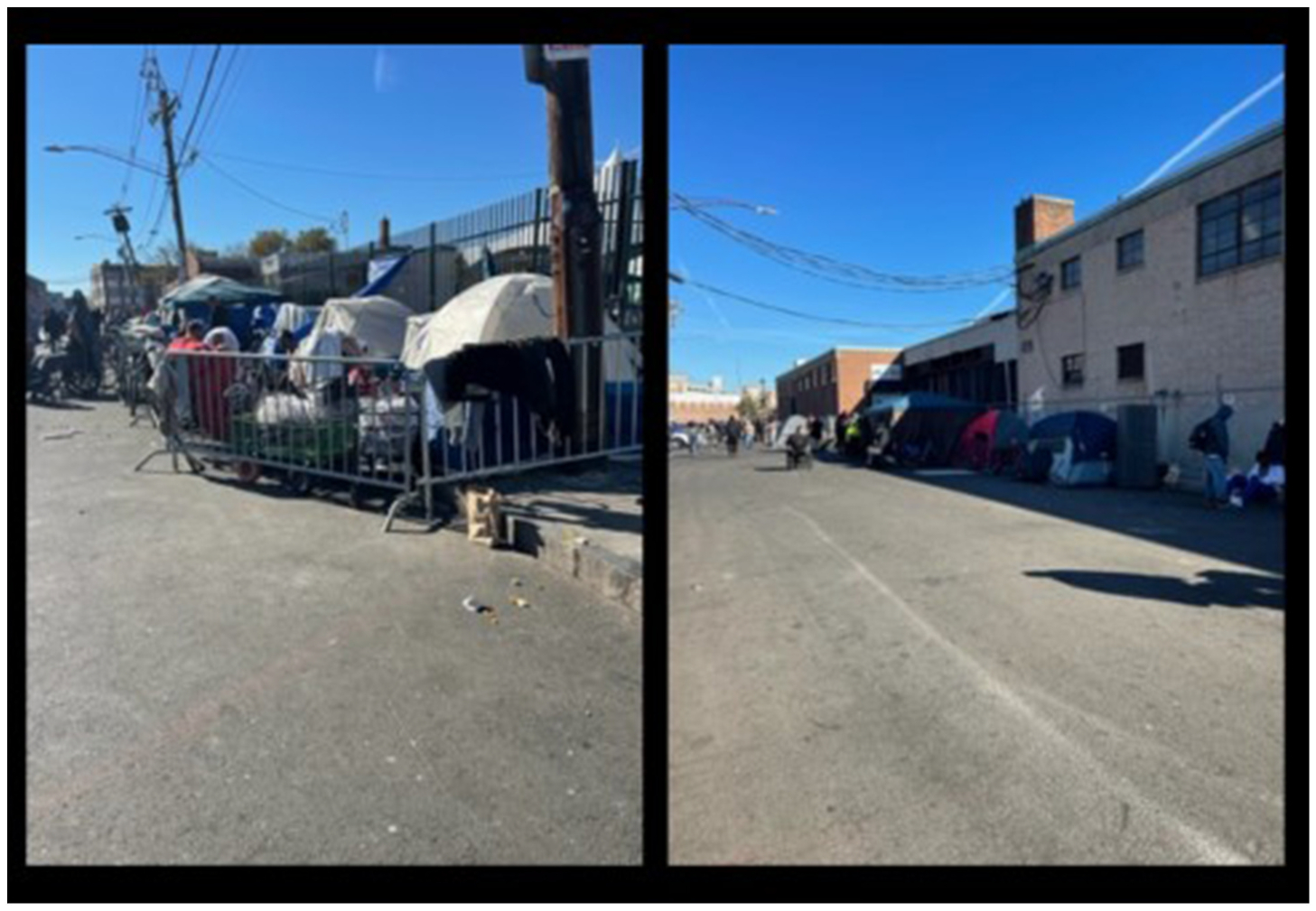
Tent encampment on Atkinson Street in Mass and Cass, November 11th, 2022.

All surveyed study participants previously resided in the Mass and Cass area (e.g., shelter) or encampments (see Fig. 1 below) then relocated to one of the seven LTTH locations in Boston neighborhoods. In January of 2022, the first LTTH location opened. To our knowledge, at the start of our data collection, the longest a person had lived at any LTTH site was about a month and a half, with some residents having just moved in that month.

Our photo-ethnographic analysis highlights how LTTH residents engaged in placemaking to claim their transitional spaces as a means of legitimizing their political identity – someone deserving of a home. This is particularly important as residents were often provided transitional housing options as a means of removing them from the encampment. That said, some, LTTH sites were located farther from the encampment, which disrupted access to clinical services or medication but also distanced them from the proximate drug use and violence at Mass and Cass. Our analysis highlights three subthemes derived from photo analysis and ethnographic observations: 1). Security and privacy 2). Personalization and self-care and 3). Resistance to dehumanization. Photographs and observations revealed how LTTH site infrastructure facilitated resident autonomy and community, while field observations uncovered how unique harm reduction facilitated placemaking.

### 3.1. Security and privacy

During our ethnographic observations, LTTH residents frequently reported concerns surrounding theft. The extent of privacy and security afforded to residents varied by site. In some cases, the physical infrastructure of the LTTH site itself facilitated security measures. For example, at Site B, residents had locking doors. For many residents, this represented a dramatic change from encampment life, where privacy and protection were minimal and residents could be subject to theft, disruption or removal at any time. However, at sites without locking doors or other facilitators of personal privacy, LTTH residents often found ways to maximize their own sense of security. Some residents implemented their own security systems – ranging from basic to highly sophisticated approaches.

**Fig. 2.**
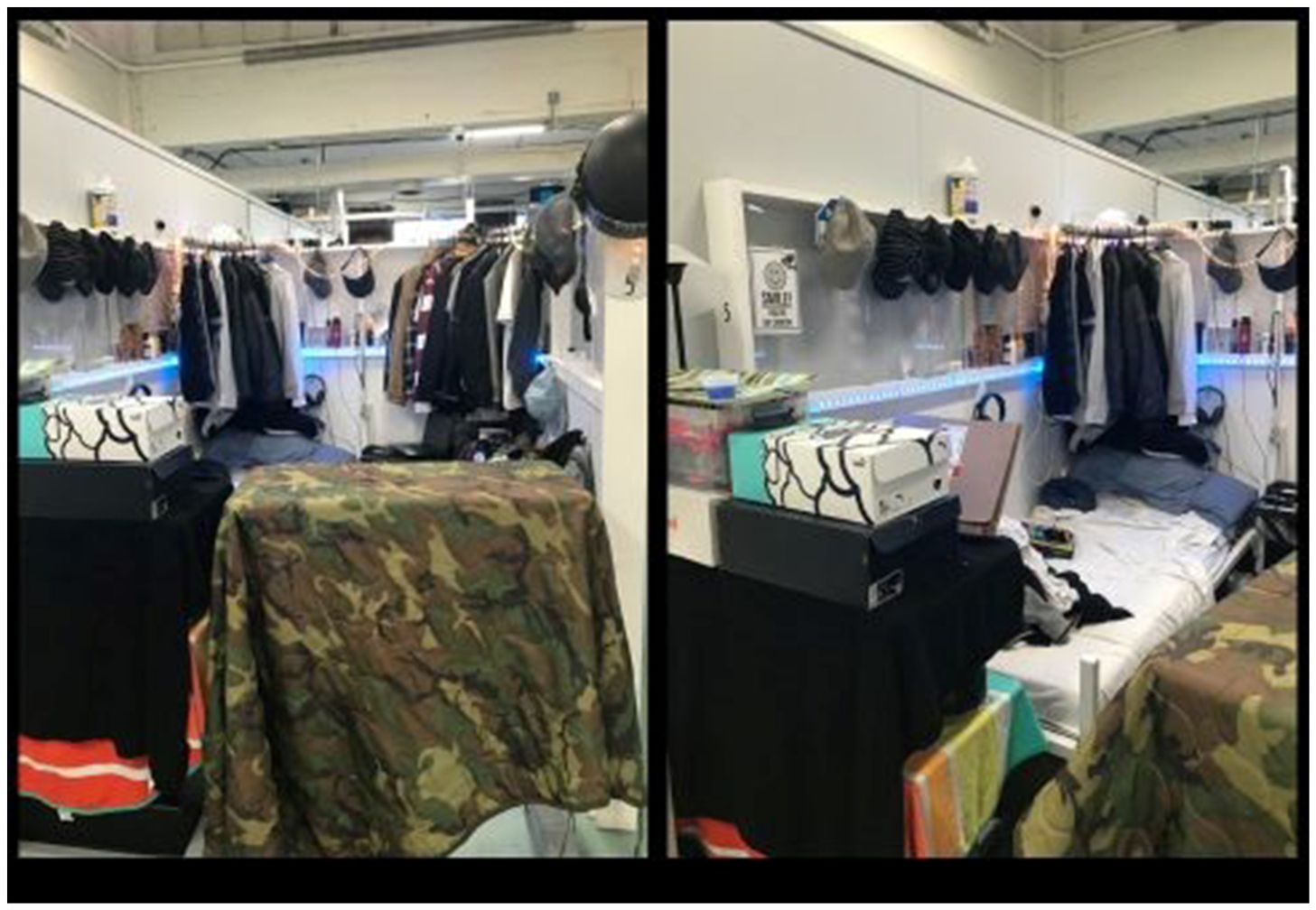
Resident cubicle at Site A, December 22nd, 2023.

For instance, Site A had residents sleep in semi-private cubicles. While these cubicles lacked doors, many residents created their own makeshift door/barricade, blocking the entrance to their cubicle with household items like chairs or suitcases, sometimes covering their individual entrances with blankets (see Fig. 2). The barricades created by residents not only provided privacy but also served as an extra layer of security to deter resident-on-resident theft. One resident outfitted his cubicle with both a barricade and a comprehensive, self-installed security system. The system included multiple security cameras which he reported sent recorded video output to a personal laptop, as well as a sign, reminiscent of those which often accompany commercial security systems, which read “*Smile, you’re on camera*” (see Fig. 2). When a study staff member asked about his security setup, the resident informed them that he had three cameras, but that the location of one of them was secret as an extra protection against theft. Through the construction of makeshift security systems, residents mitigate the risks of communal living and assert an active role in protecting their personal possessions. Coming from the encampment setting where ownership of their belongings was continuously undermined by theft, policing, confiscation, and ‘street cleanings’, self-made security measures assert a new identity as someone who has legitimate claims to both personal space and personal property.

**Fig. 3.**
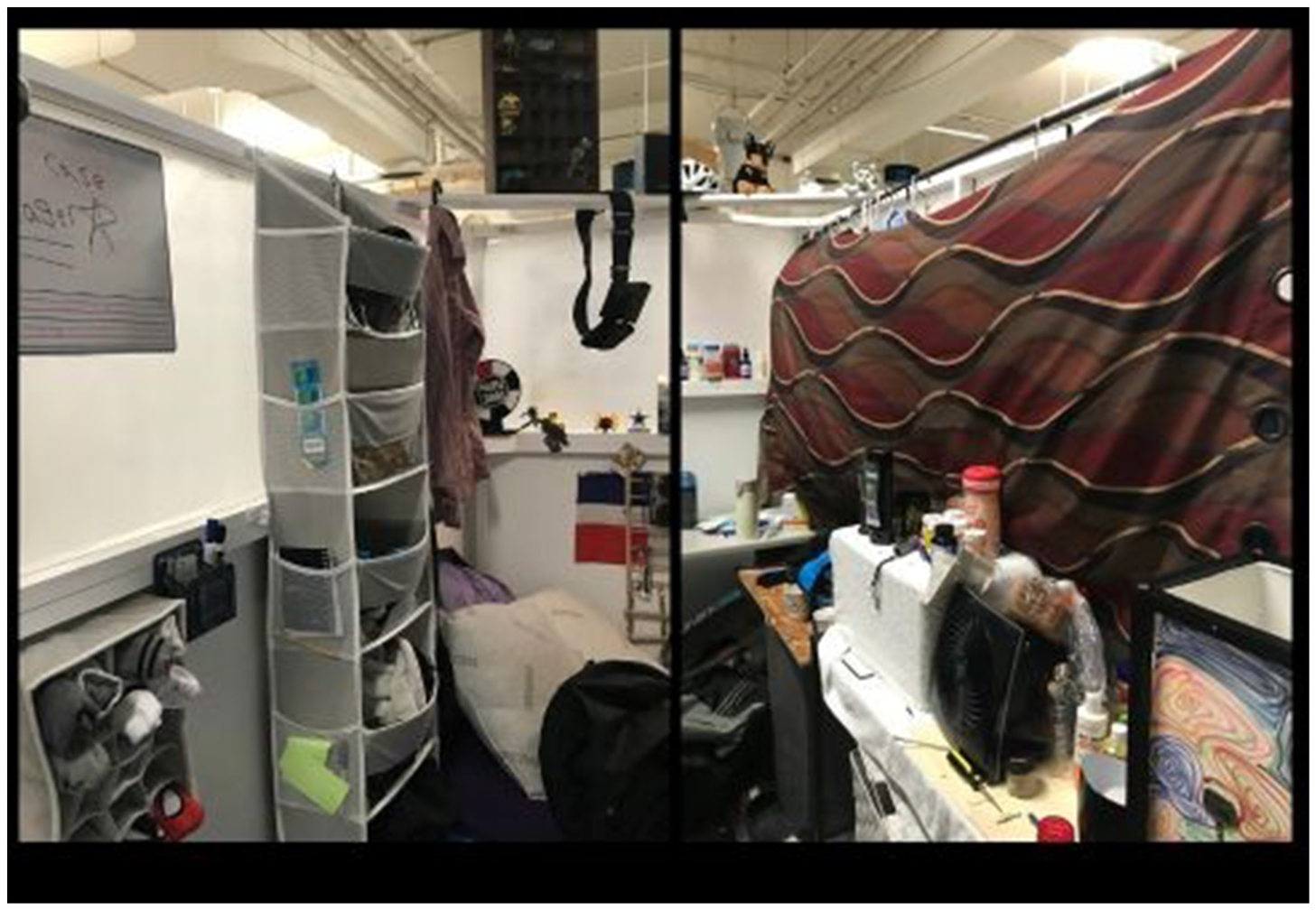
Two different resident cubicles at Site A, December 22nd, 2023.

Additionally, residents maximized storage capacity through self-made storage space and physical modification of existing LTTH infrastructure. Each LTTH site differed in the amount of storage space provided to residents, ranging from entire closets to small shelves. As a result, the ways residents organized their belongings to optimize storage often varied. In single-occupancy rooms, space allowed for residents to set personal belongings to the sides of their beds and distribute them more evenly around their rooms. At the sites where residents shared space with others, whether through semi-open cubicles or shared bedrooms, belongings were often stored vertically, above or next to their beds. Residents created makeshift closets in efforts to conceal their belongings. Fig. 3 (right) captures a resident cubicle where the resident used the space between the back of his cubicle and the back wall as extra storage space. This same resident also created his own closet by assembling a bar for hanging clothes and covering it with a blanket. Also seen in Fig. 3, residents created their own shelving to overcome limitations to provided storage space while legitimizing and maximizing the allotted personal space.

**Fig. 4.**
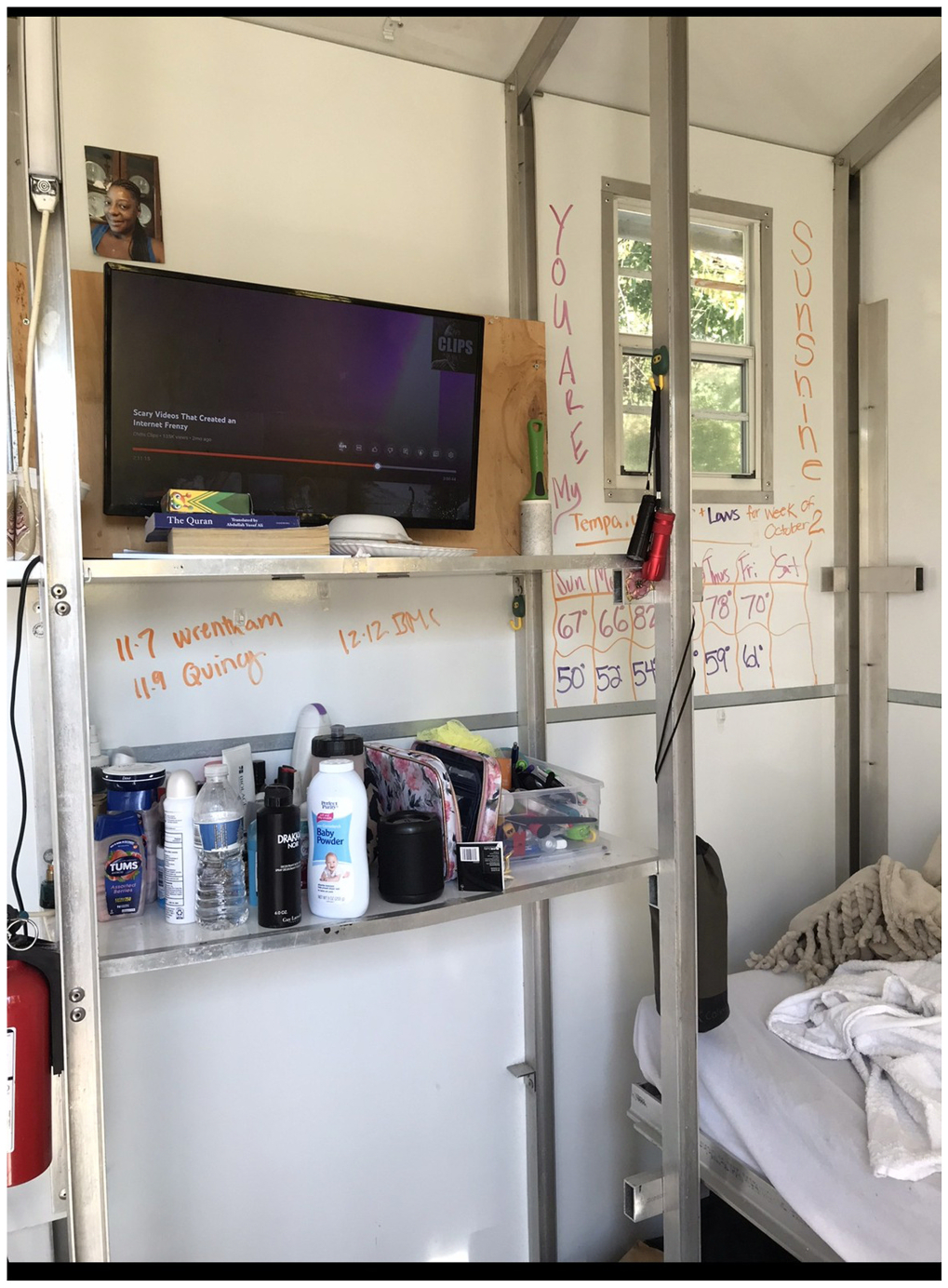
Homemade shelving in a resident’s tiny house at Site B, December 28th, 2023.

Many LTTH residents came to the sites following an extended stay at encampments subjected to rigorous street cleaning programs and intermittent police sweeps, requiring them to limit and monitor their belongings or risk losing them. LTTH sites provided these residents new opportunities for collection and protection of belongings. In many cases, residents needed to modify their living spaces in order to store and secure their newfound items. This storage and collection of belongings became another way that LTTH residents asserted their own permanence and legitimacy within the LTTH sites. In doing so, residents claimed space as if they were afforded their own apartment or living space. Residents created and reinforced storage spaces, making semi-permanent structures which ensured those belongings are protected (see Fig. 4). While ‘collecting’ and accumulation of belongings is also commonplace in street homelessness, the practices of accumulation at LTTH sites meaningfully differed in that belongings were organized and stored with a sense of permanence. Even in particularly chaotic arrangements of items, researchers observed efforts to arrange or store belongings in a systematic way, implying an expectation that the items could be returned to at a given moment. These behaviors appeared distinct from compulsive hoarding, as items were stored with a purpose. This difference is particularly stark given the examples of those residents who created their own closets or shelves (see Fig. 3).

**Fig. 5.**
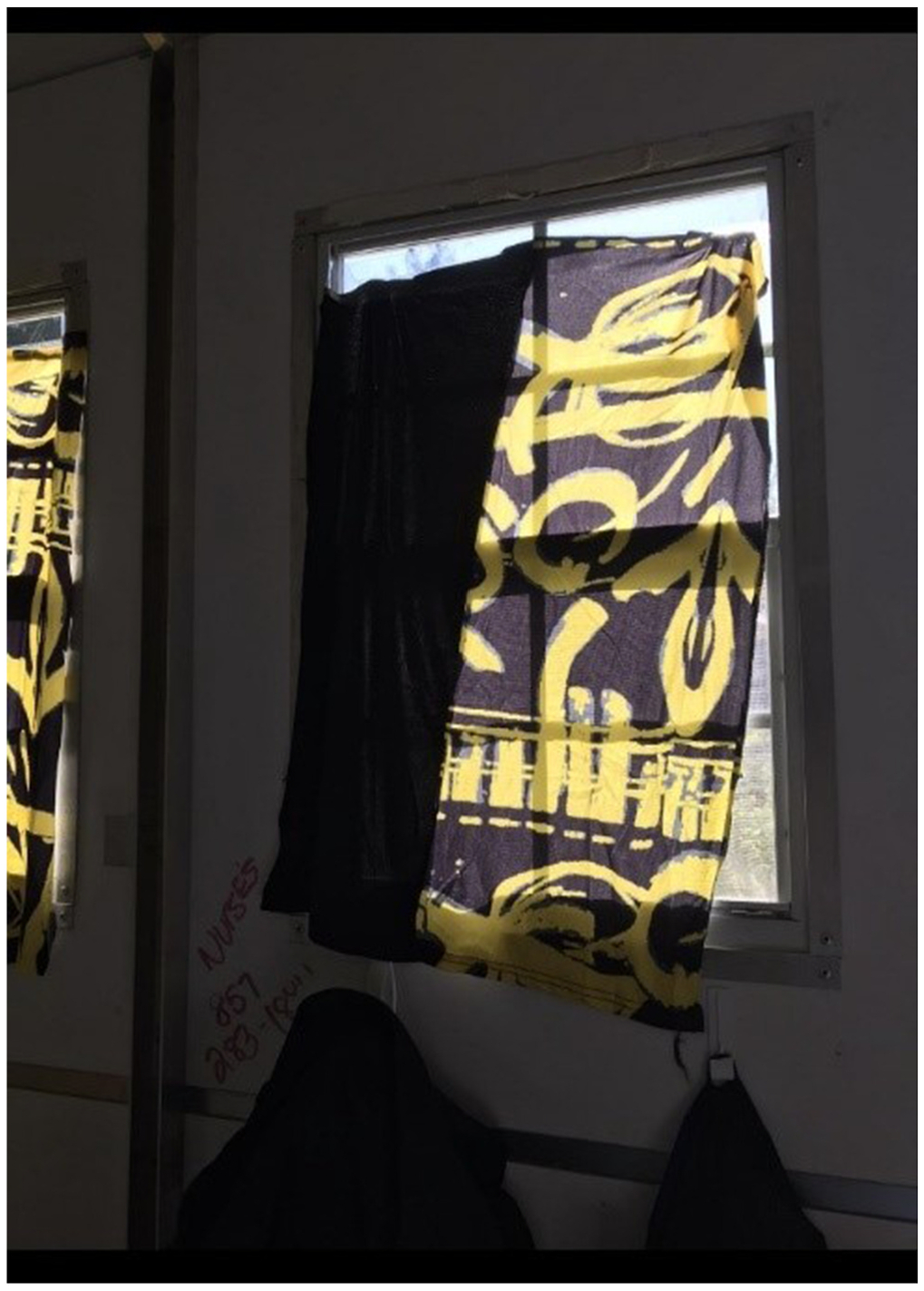
Window in a resident’s tiny house at Site B, December 28th, 2023.

Residents found other ways to maximize personal privacy within the infrastructural confines of their personal living quarters. Site B provided residents with windows into their rooms, but no curtains. As such one resident created their own curtain to cover their windows to ensure an additional layer of privacy (see Fig. 5).

Through modifying their physical space, residents asserted a right to privacy within infrastructural constraints by exercising their autonomy to address unmet structural needs. Given that many of these residents transitioned directly from the Mass and Cass encampment, where police involvement and regular ‘street cleanings’ prevented establishment of privacy structures, these personal self-directed safety measures are particularly noteworthy. Existing infrastructure at LTTH sites, combined with policies which facilitated autonomy, afforded residents the opportunity to assert a right to privacy through innovative approaches. While these opportunities to modify one’s own space were not necessarily in line with the possibilities offered by permanent housing, they still emerged as significantly different from those present at more traditional shelters. Staff at LTTH sites often tolerated or actively encouraged modifications to residents existing space as long as these safety measures were not impeding resident safety (e.g. blocking hallways or emergency exits). In these instances, staff and LTTH residents engaged in a process of collaborative placemaking, where adaptive policies (or willingness not to enforce site policies) assisted in the development of a sense of autonomy which in turn served to assist in promoting privacy for residents.

### 3.2. Personalization and self-care

**Fig. 6.**
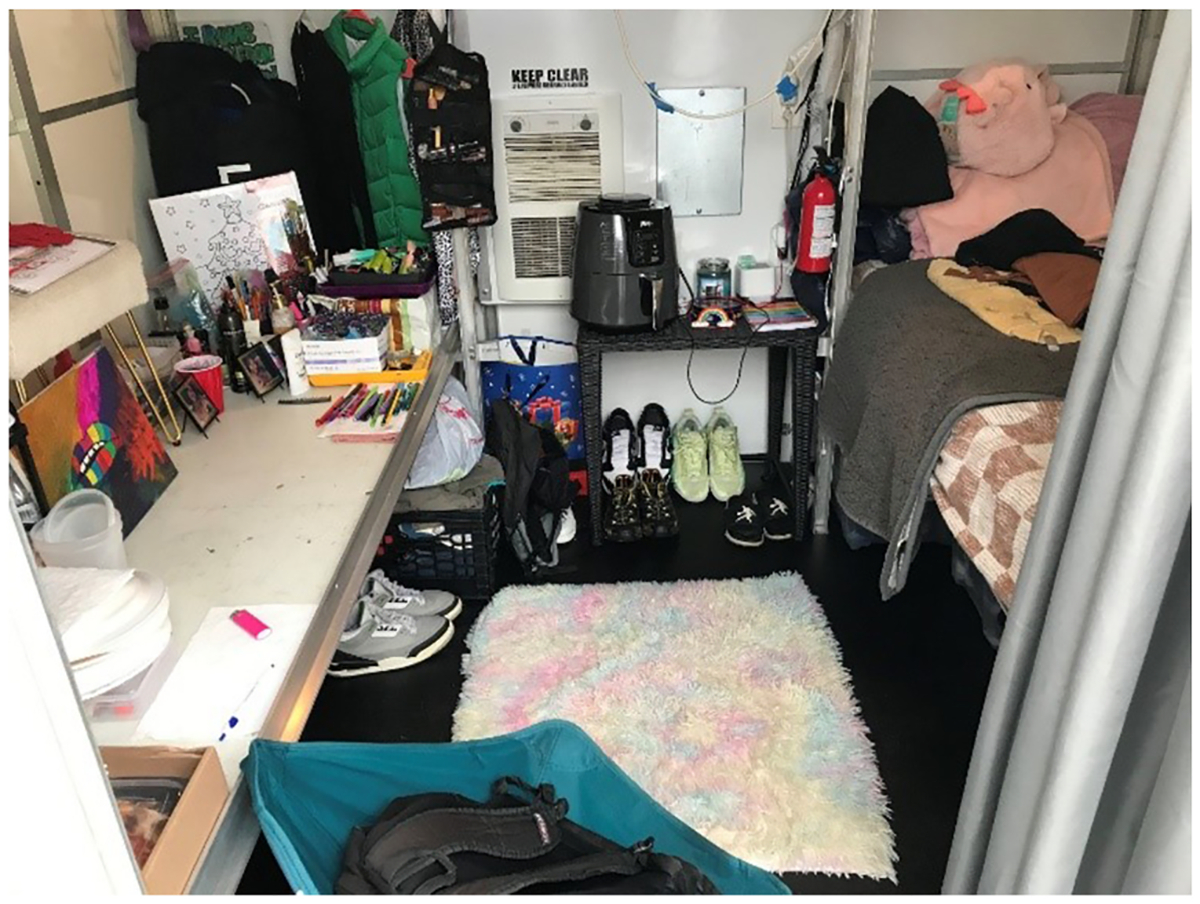
Resident’s tiny house room at Site B, May 1, 2024.

All photographs taken displayed at least some level of personalization, showing how residents reclaim or redevelop identities in their space. Residents often engaged in a variety of tactics to personalize their spaces. Some residents did so through artwork, a few even displaying their own self-created artwork such as paintings and drawings. Some photographs captured evidence of art-in-process, such as paints, markers, and journals. Fig. 6 depicts one resident’s tiny home cottage. Next to her bed is a table full of art supplies and a painting of lips. When a study staff member inquired about the art supplies and painting, the resident stated that she enjoys drawing in her free time. Together with her partner, she painted and created artwork to decorate their space. She described this hobby as both an expression of creativity and a therapeutic activity. The stability of LTTH permitted her the time and stability to engage in her hobbies, while the infrastructure provided a space for her to create and display her artwork.

Also seen in Fig. 6 is an air fryer, which the resident used to prepare her own meals independently. At the time this picture was taken, this resident proudly explained that they were cooking chicken wings for lunch. At four sites, residents were able to store food in their rooms. At two of these sites, many residents outfitted their rooms with small appliances for personal food preparation allowing them to eat whenever they pleased, a stark difference from conventional shelter environments. This highlights not only the independence afforded to residents, but also their acts of preparation for future independent living. Living in this transitional space after spending significant time unhoused, residents are given new opportunities to practice healthy life skills associated with independent living. Some residents, having been unhoused since a young age, had never been afforded the opportunity to develop these essential life skills. LTTH policies and staff facilitate this personal growth through offering residents’ flexible opportunities to organize and utilize space. Additionally, residents’ spaces often contained items used to practice ‘self-care’, such as medications, personal hygiene products, and makeup. Several residents across different sites kept cleaning supplies such as make up and sanitizing wipes and shampoos. Notably, many residents at the female-only sites also stored their makeup items neatly on table or shelf space (see Fig. 6 (right)). Evidence of self-care beyond basic hygiene needs, the storage of makeup contributes to a sense that residents are given space for self-expression.

**Fig. 7.**
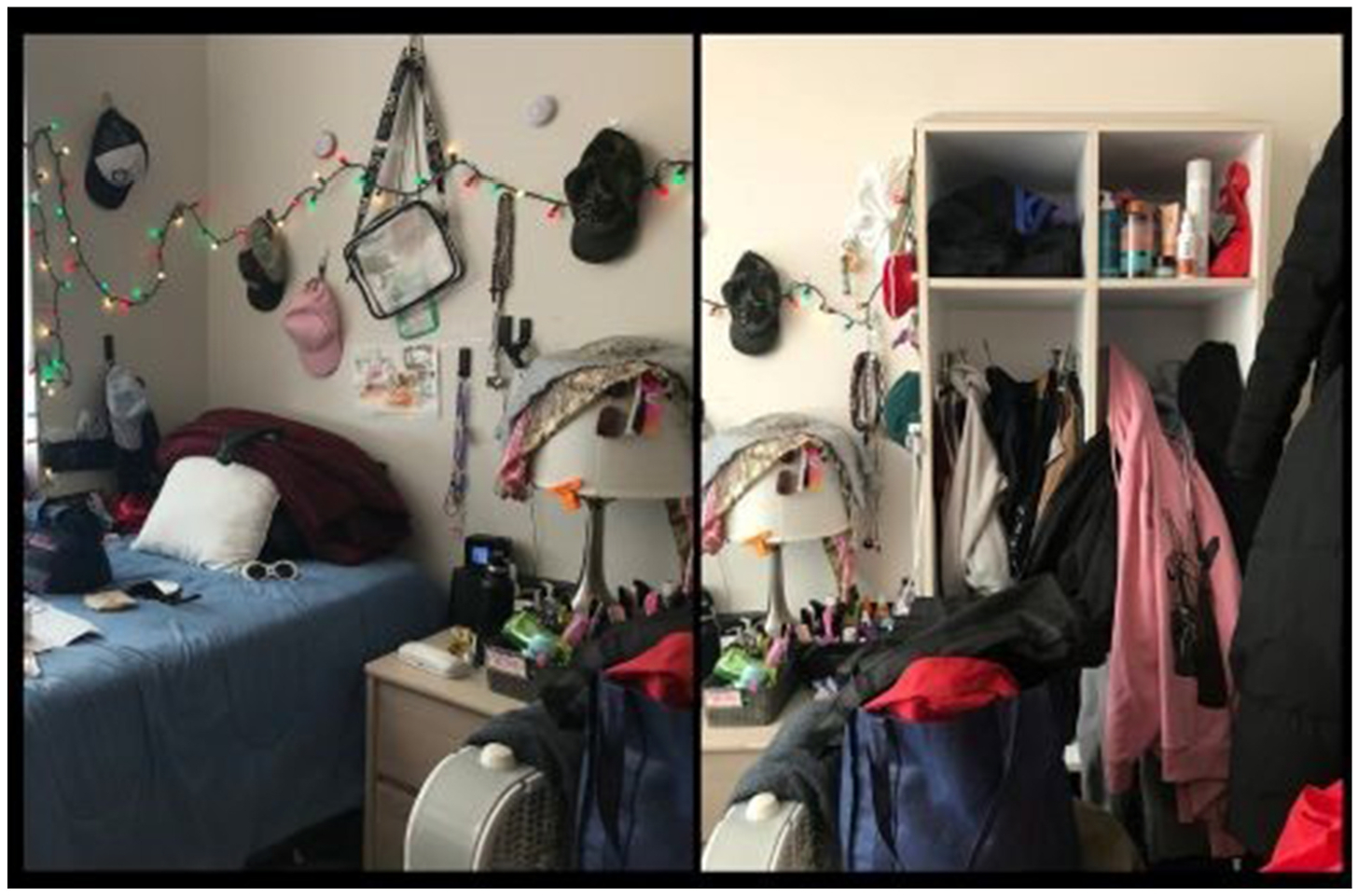
Shared resident room at Site D, April 12, 2023.

Other LTTH residents had their own personal collections on display, varying from hats, sunglasses, shoes, toys and figurines. Fig. 7 depicts one resident’s collection of hats and sunglasses, as well as miscellaneous artwork. The practice of placemaking within LTTH allows for forms of ownership and accumulation that were previously not possible in the context of encampment life or traditional shelter environments and facilitated the forming or reclaiming of personal identity. Residents’ collections of trinkets, memorabilia, and hobby objects reinforced their identities as people with histories and interests beyond just their housing status or substance use.

### 3.3. Resistance

It is not uncommon for marginalized groups like the unhoused community to contest their right to space through deliberate modification of their environment (Douglas, 2023; Wright, 1997). While this resistance often plays out on the street in the form of struggles with businesses, city officials or law enforcement over formation of encampments and accumulation of belongings in public spaces (Protschky, 2023; Wuthmann, 2023), the resistance practiced at LTTH sites is unique in that it appears to represent the intention of establishing a right to permanent space and belonging. While the LTTH sites were created as a means to rapidly re-house and relocate people from the Mass and Cass encampment, in many instances, transitional housing became the norm, with some residents stuck within low-threshold housing for indefinite periods of time due to barriers such as clearing warrants, obtaining identification records or simply bureaucratic delays in the provision or availability of long-term housing. Residents responded to these perceived bureaucratic failures not just through complaints, but also physical modifications to their provided space.

**Fig. 8.**
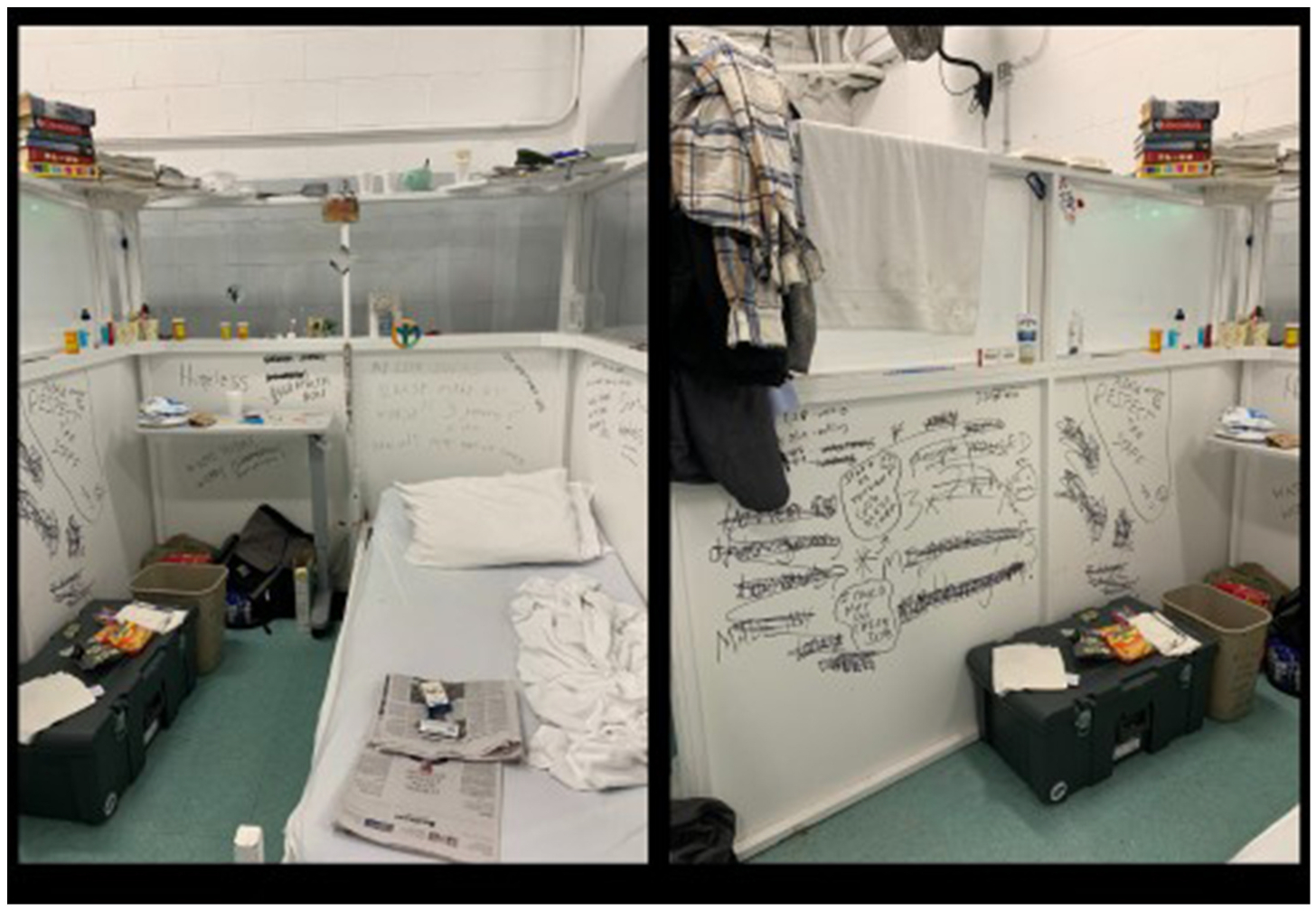
Walls of a resident’s cubicle at Site A, July 11, 2024.

Fig. 8 depicts an instance where an LTTH resident wrote on the walls of their cubicle. The resident inscribed phrases such as “*hopeless*” and “*My life sucks!!! Please make it worse. 3 years of this*?’, expressing frustrations about the amount of time spent within their transitional housing space. On the left-hand side of the image, a series of dates on which he did not receive long-term housing are marked as “*nothing*”. When research staff spoke with the resident, he revealed that many of the other statements inscribed on the wall came from songs, including a piece from “Smuggler’s Blues” by Glenn Frey, which expresses remorse over a drug-related murder, and “*Richard Hung Himself*” by D.I., a song recounting a suicide fueled by hopelessness and heroin use. Other statements on the wall expressed his inability to obtain public health insurance, writing out the exact sums of his detox bills. Other markings requested that others “*Please Respect the Staff*” and insisted that “*Staff is patient, God Bless them, I could not do their job*”, illustrating that his frustrations were not primarily directed at staff but were instead an attempt to assert agency and legitimacy while also communicating his dissatisfaction surrounding the process of obtaining permanent housing.

**Fig. 9.**
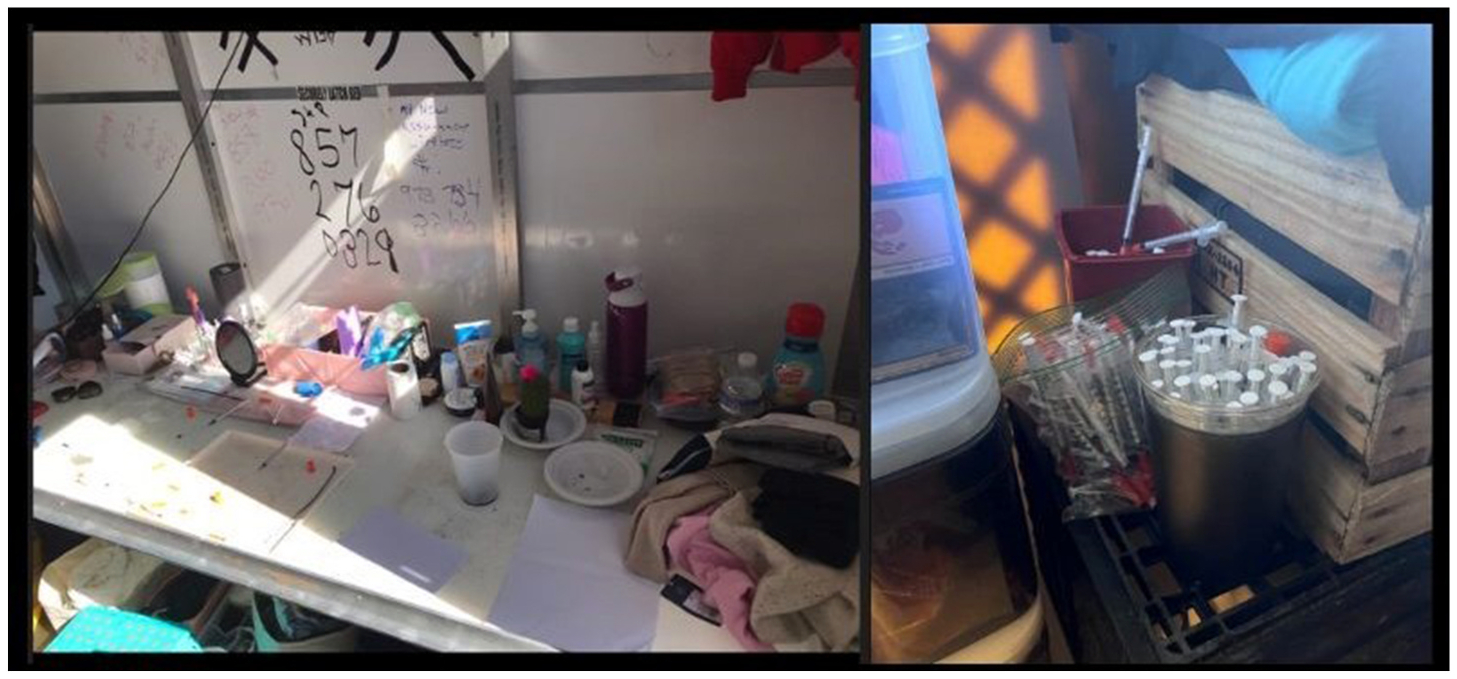
Drug use space inside a tiny house at Site B, March 14th, 2024.

Residents also engaged in resistance through their organization and preparation of recreational drugs. The majority of LTTH residents actively engaged in regular drug use. While no LTTH sites *officially* permitted drug use on-site (primarily due to local laws and building ordinances), about half the sites were tolerant of on-site substance use, especially when out of sight of others, in private rooms. There, many residents constructed personal drug use stations within their private spaces. In these instances, some residents created their own injection stations, laying out materials for safe and hygienic drug use preparation such as cookers, cottons, and syringes (see Fig. 9). In addition to facilitating safer drug use habits, the ability to create these drug use preparation stations signaled a right to autonomy within one’s private space, even when engaging in stigmatized behavior like drug use.

## 4. Discussion

Our findings must be understood in the context of mass displacement and encampment clearings in a large metropolitan city. Almost all residents in our study spent some portion of their life living in the Boston’s Mass and Cass encampment and their placement into LTTH occurred in the context of a citywide efforts to “clear” the streets following the declaration of a “state of emergency” (Dwyer, 2021). Thus, LTTH housing came about not as an effort to assert notions of housing as a human right, but as a tool through which the city could rapidly relocate members of a highly visible and contentious unhoused population without incurring significant political and community backlash. The rapid development of these sites, often through the modification or reuse of existing spaces like converted hotels or office space, created the necessary infrastructure to provide low-threshold transitional housing options. This, in turn, allowed programs to place unhoused people into housing environments that offered support, resources and afforded them a level of privacy and autonomy not typically received in conventional shelters. However, the trajectory and sustainability of these LTTH sites remains in question, as many have been shuttered or are facing threats of defunding. Even so, our photo ethnographic analysis found that LTTH residents, like many other communities of unhoused people (Wright, 1997), engaged in intentional placemaking efforts as an act of resistance, asserting their presence and identity in an environment that sought to remove them from public space. Creative placemaking is ultimately an exercise in belonging (Bedoya, 2013). Residents reinforced their sense of belonging through their placemaking practices, becoming active participants in their own environment and creatively devising ways to assert new forms of autonomy and identity. Despite the cynical circumstances of their original relocation, both staff and residents of LTTH sites dared to “trust in the possibility of a beloved place” and assert their right to play their “own significant part in the making of such places” (Bennett, 2000; Schneekloth and Shibley, 1995).

Placemaking theorists argue that self-modification of one’s environment often reflects one’s needs and wants (Proshansky et al., 1983) and that places themselves are a primary means by which we stabilize our identities in that world (Dovey, 2010). The infrastructural modifications made by LTTH residents are key to understanding their desires for permanent stable housing. From makeshift closets to home-grown security systems, the modifications enacted by residents demonstrate their desire for autonomy, safety, and privacy within the context of transitional housing. These needs may emerge from residents’ recent experiences with mass displacement, coming into LTTH from a variety of living situations (such as shelters with restrictive non-LTTH policies, tents, couch surfing or on the street) where these offerings were scarce or even non-existent. Due to heavy policing, former encampment residents had limited privacy and autonomy over their space. Following their placement into LTTH, residents were able to utilize the resources available to them to obtain the autonomy, safety, and privacy that were previously unattainable.

Further, resident placemaking facilitated the construction of a non-stigmatized personal identity as residents experienced meaningful identity transformations throughout their time in LTTH locations. As unhoused people who use drugs, many LTTH residents occupy a deeply stigmatized identity, one which is often construed as inherently outside of and a threat to the wider community (Zigon, 2019). As residents at LTTH sites transitioned from life on the street into transitional housing sites they experienced important role shifts away from previously held identities (e.g., being unhoused) by embodying newly formed identities within housing. This process of role exit highlighted by (Ebaugh, 1988) demonstrates a process through which residents established new identities within the context of transitional housing, thereby attempting to create distance from their stigmatized former selves. Research has shown the importance of identity transformations both personally and socially for individuals who experienced homelessness indicating that these shifts are important for identity reconstruction (Boydell et al., 2000; McCarthy, 2013; Preece et al., 2020). Our findings mirror observations that placemaking can be used to challenge externally imposed, stigmatizing identities (Bedoya, 2013) and as a means to establishing a new sense of self through performative social measures (Goffman, 1959, 1963). Through efforts to create safety measures for themselves and others, LTTH residents resist narratives that depict people who use drugs as selfish and unwilling to look out for one another. The response of constructing self-directed safety measures is common practice within any type of housing environment (Ivsins et al., 2022; Briggs et al., 2009; Kerman et al., 2023) and LTTH sites permitted these innovative approaches with collaborative placemaking (Douglas, 2023) facilitated by site staff, amidst a lack of consistent security infrastructure. Additionally, through personalizing their spaces, residents resist reduction of their identity to that of merely someone who uses drugs or who is unhoused demonstrating their own sense of personal identity and self-care through self-expression. Their creation of safe and organized drug use spaces implicitly challenges dominant expectations that they must become abstinent before obtaining a place of their own. In this way, “placemaking may contribute to transforming community narratives,” with LTTH residents working to subvert negative assumptions and stigma from the wider community (Toolis, 2017).

Place, agency, and identity do not form distinctly, but are instead co-constituted. That is, having one’s own space facilitates agency and the formation of particular identities, while identity and agency shape how people use, engage with, and create place. Each of these three elements worked together, dynamically, coalescing in a placemaking process which affirmed residents’ right to permanent housing. LTTH residents “use space … to create, present, sustain a personal identity tied to place and to contest alternative meanings, degradations, and stigma” (Gotham and Brumley, 2002). LTTH staff and the sites’ unique harm reduction-oriented policies (Zaragoza et al., 2025) facilitated the development of this process through affording residents the ability to modify their own space, tolerating activities and practices forbidden in traditional housing and shelter settings, and providing ready access to supplies and services that facilitated placemaking. It is debatable whether the provision of housing without a harm reduction orientation would have inspired similar emergent themes of placemaking following a large encampment disbursement. However, LTTH provided the necessary infrastructure that could facilitate placemaking processes.

Being relatively new interventions, it is difficult to measure the effectiveness of LTTH housing, whether it be in improving health outcomes, decreasing homelessness, or other potential goals. However, it is important to note that the harm reduction orientation of each site focused on the provision of resources and implementation of policies designed to reduce drug related harms. In particular, practices like room checks, sedation monitoring, providing naloxone, and maintaining on-site nursing staff aimed to reduce overdose rates among residents. In a city with a significant overdose rate, the importance of these interventions cannot be overstated. While this paper focuses on the process of placemaking among LTTH residents, we do not want to lose sight of the fact that LTTH housing was designed with the safety of PWUD in mind. While we are unable to draw any strong conclusions about the effectiveness of these measures in preventing drug-related harms. more research is needed to better understand the full effect of these innovative housing models on resident safety, behaviors, and health, and the role of placemaking in this process.

### 4.1. Limitations

There are several important limitations in our study. First, our study used exploratory photographic methods to understand how residents utilized LTTH spaces. The study team took photographs with permission from staff and residents, but photographs were not taken systematically, targeting specific angles, locations or items. Instead, the photographs explore how spaces were constructed and utilized by residents within LTTH. Second, while researchers recorded field notes for some photographs, noting residents’ statements as to why spaces were structured in particular ways, this was not always possible and some photographs were interpreted solely by the study team. Third, due to privacy concerns and institutional policy, our team was only able to photograph personal spaces from four of the seven sites. Also, due to privacy concerns, resident demographic information and survey results were kept completely separate from photographs, preventing us from including this information in our analysis. Future research within LTTH locations should consider participatory methods like photovoice that would allow residents to take photographs on their own, engaging them in dialogue about how they see and utilize spaces. The use of this methodology would provide more context into how residents conceptualize and enact placemaking within the context of transitional housing locations.

## 5. Conclusion

This analysis opens critical windows to understanding and investigating the ways that LTTH residents establish belonging, forge identity, and assert a right to permanency, even within impermanent transitional spaces. Our findings show that amidst mass relocation efforts, residents made concerted efforts to engage in placemaking within LTTH sites. These efforts were representative of ensuring rights to privacy, self-care, personalization and resisting stigmatizing political narratives about their life circumstances as they transitioned from street-homelessness into transitional housing models. Our research is situated within the broader context of placemaking literature and highlights the importance of these unique spaces in constructing non-stigmatized identity and facilitating a sense of belonging and purpose. While some research has focused on placemaking among unhoused populations (Vasudevan, 2015; Fathi et al., 2025), less research has extensively explored place-making within transitional housing structures like LTTH. As a mechanism of societal change with profound human impact, LTTH models in particular are investments in a healthier, more stable, and housed future for a vulnerable population. Future studies should further explore the role that placemaking within similar housing communities plays in shaping new identities and facilitating assertions of belonging and permanency among unhoused people. Additionally, future research should continue to explore the idea of placemaking as it pertains to how residents engage with space and place in transitional housing, how residents utilize transitional housing spaces to secure more permanent housing options, and how effects differ for placemaking in transitional in contrast to permanent housing settings.

## Data Availability

The datasets used and/or analyzed during the current study are available from the corresponding author on reasonable request.

## Data availability

Data will be made available on request.

## GLOSSARY

LTTH: Low Threshold Transitional Housing
PWUD: People Who Use Drugs
MOUD: Medications for Opioid Use Disorder
